# Efficient Noninferiority Testing Procedures for Simultaneously Assessing Sensitivity and Specificity of Two Diagnostic Tests

**DOI:** 10.1155/2015/128930

**Published:** 2015-08-20

**Authors:** Guogen Shan, Amei Amei, Daniel Young

**Affiliations:** ^1^Epidemiology and Biostatistics Program, Department of Environmental and Occupational Health, School of Community Health Sciences, University of Nevada Las Vegas, Las Vegas, NV 89154, USA; ^2^Department of Mathematical Sciences, University of Nevada Las Vegas, Las Vegas, NV 89154, USA; ^3^Division of Health Sciences, University of Nevada Las Vegas, Las Vegas, NV 89154, USA

## Abstract

Sensitivity and specificity are often used to assess the performance of a diagnostic test with binary outcomes. Wald-type test statistics have been proposed for testing sensitivity and specificity individually. In the presence of a gold standard, simultaneous comparison between two diagnostic tests for noninferiority of sensitivity and specificity based on an asymptotic approach has been studied by Chen et al. (2003). However, the asymptotic approach may suffer from unsatisfactory type I error control as observed from many studies, especially in small to medium sample settings. In this paper, we compare three unconditional approaches for simultaneously testing sensitivity and specificity. They are approaches based on estimation, maximization, and a combination of estimation and maximization. Although the estimation approach does not guarantee type I error, it has satisfactory performance with regard to type I error control. The other two unconditional approaches are exact. The approach based on estimation and maximization is generally more powerful than the approach based on maximization.

## 1. Introduction

Sensitivity and specificity are often used to summarize the performance of a diagnostic or screening procedure. Sensitivity is the probability of positive diagnostic results given the subject having disease, and specificity is the probability of a negative outcome as the diagnostic result in the nondiseased group. Diagnostic tests with high values of sensitivity and specificity are often preferred and they can be estimated in the presence of a gold standard. For example, two diagnostic tests, the technetium-99m methoxyisobutylisonitrile single photon emission computed tomography (Tc-MIBI SPECT) and the computed tomography (CT), were compared for diagnosing recurrent or residual nasopharyngeal carcinoma (NPC) from benign lesions after radiotherapy in the study by Kao et al. [1]. The gold standard in their study is the biopsy method. The sensitivity and specificity are 73% and 88% for the CT test and 73% and 96% for the Tc-MIBI SPECT test.

Traditionally, noninferiority of sensitivity and specificity between two diagnostic procedures is tested individually using the the McNemar test [2–6]. Recently, Tange et al. [7] developed an approach to simultaneously test sensitivity and specificity in noninferiority studies. Lu and Bean [2] were among the first researchers to propose a Wald-type test statistic for testing a nonzero difference in sensitivity or specificity between two diagnostic tests for paired data. Later, it was pointed out by Nam [3] that the test statistic by Lu and Bean [2] has unsatisfactory type I error control. A new test statistic based on a restricted maximum likelihood method was then proposed by Nam [3] and was shown to have good performance with actual type I error rates closer to the desired rates. This test statistic was used by Chen et al. [8] to compare sensitivity and specificity simultaneously in the presence of a gold standard. Actual type I error rates for a compound asymptotic test were evaluated on some specific points in the sample space. It is well known that the asymptotic method behaves poorly when the sample size is small. Therefore, it is not necessary to comprehensively evaluate type I error rate [9–14].

An alternative to an asymptotic approach is an exact approach conducted by enumerating all the possible tables for given total sample sizes of diseased and nondiseased subjects. The first commonly used unconditional approach is a method based on maximization [15]. In the unconditional approach, only the number of subjects in the diseased and nondiseased group is fixed, not the total number of responses from both groups. The latter is considered as the usual conditional approach by treating both margins of the table as fixed. The *p* value of the unconditional approach based on maximization is calculated as the maximum of the tail probability over the range of a nuisance parameter [15]. This approach has been studied for many years and it can be conservative due to a smaller actual type I error rate as compared to the test size in small sample settings. One possible reason leading to the conservativeness of this approach is the spikes in the tail probability curve. Storer and Kim [16] proposed another unconditional approach based on estimation which is also known as the parametric bootstrap approach. The maximum likelihood estimate (MLE) is plugged into the null likelihood for the nuisance parameter. Other estimates may be considered if the MLE is not available [7]. Although this estimation based approach is often shown to have type I error rates being closer to the desired size than asymptotic approaches, it still does not respect test size.

A combination of the two approaches based on estimation and maximization has been proposed by Lloyd [4, 17] for the testing of noninferiority with binary matched-pairs data, which can be obtained from a case-control study and a twin study. The *p* value of the approach based on estimation is used as a test statistic in the following maximization step. It should be noted that there could be multiple estimation steps before the final maximization step. The final step must be a maximization step in order to make the test exact. This approach has been successfully extended for the testing trend with binary endpoints [5, 18]. The rest of this paper is organized as follows.  Section 2 presents relevant notation and testing procedures for simultaneously testing sensitivity and specificity. In Section 3, we extensively compare the performance of the competing tests. A real example is illustrated in Section 4 for the application of asymptotic and exact procedures.  Section 5 is given to discussion.

## 2. Testing Approaches

Each subject in a study is evaluated by two dichotomous diagnostic tests, *T*_1_ and *T*_2_, in the presence of a gold standard. Suppose each subject, either diseased or nondiseased, was already determined by the gold standard before performing the two diagnostic tests. Within the diseased group, *n*_*ij*_ (*i* = 0,1; *j* = 0,1) is the number of subjects with diagnostic results *T*_1_ = *i* and *T*_2_ = *j*, where *T*_*k*_ = 0 and *T*_*k*_ = 1 represent negative and positive diagnostic results from the *k*th test (*k* = 1,2), respectively, with *p*_*ij*_ being the associated probability. The total number of diseased subjects is *n* = *n*_00_ + *n*_10_ + *n*_01_ + *n*_11_. Similarly, *m*_*ij*_ (*i* = 0,1; *j* = 0,1) is the number of subjects with diagnostic results *T*_1_ = *i* and *T*_2_ = *j* in the nondiseased group, *q*_*ij*_ is the associated probability, and *m* = *m*_00_ + *m*_10_ + *m*_01_ + *m*_11_ is the total number of nondiseased patients. Such data can be organized in a 2 × 2 × 2 contingency table (Table 1), where **N** = (*n*_00_, *n*_10_, *n*_01_, *n*_11_) and **M** = (*m*_00_, *m*_10_, *m*_01_, *m*_11_). It is reasonable to assume that the diseased group is independent of the nondiseased group.

In a study with given total sample sizes *n* and *m* in the diseased and the nondiseased groups, respectively, sensitivities of diagnostic tests *T*_1_ and *T*_2_ are estimated as ${\hat{sen}}_{1}=(n_{11}+n_{10})/n$ and ${\hat{sen}}_{2}=(n_{11}+n_{01})/n$. Similarly, ${\hat{spe}}_{1}=(m_{00}+m_{01})/m$ and ${\hat{spe}}_{2}=(m_{00}+m_{10})/m$ are specificities for *T*_1_ and *T*_2_, respectively. The estimated difference between their sensitivities is

(1) $$\begin{array}{c} {\hat{\theta }}_{sen}={\hat{sen}}_{1}-{\hat{sen}}_{2}=\frac{n_{10}-n_{01}}{n}, \end{array}$$

and the estimated difference between their specificities is

(2) $$\begin{array}{c} {\hat{\theta }}_{spe}={\hat{spe}}_{1}-{\hat{spe}}_{2}=\frac{m_{01}-m_{10}}{m}. \end{array}$$

The hypotheses for noninferiority of sensitivity and specificity between *T*_1_ and *T*_2_ are given in the format of compound hypotheses as

(3) H0:  θsen≤−δsen,or  θspe≤−δspe,

against

(4) Ha:  θsen>−δsen,θspe>−δspe,

where *δ*_sen_ and *δ*_spe_ are the clinical meaningful differences between *T*_1_ and *T*_2_ in sensitivity and specificity, *δ*_sen_ > 0 and *δ*_spe_ > 0. For example, investigators may consider a difference in sensitivity of less than 0.2 not clinically important (*δ*_sen_ = 0.2).

A test statistic for the hypotheses *H*_0_:  *θ*_sen_ ≤ −*δ*_sen_ versus *H*_*a*_:  *θ*_sen_ > −*δ*_sen_ is

(5) $$\begin{array}{c} Z_{sen}\left(\;N\;\right)=\frac{{\hat{\theta }}_{sen}+\delta_{sen}}{{\hat{\sigma }}_{sen}}, \end{array}$$

where ${\hat{\theta }}_{sen}$ is the estimated difference in sensitivities and ${\hat{\sigma }}_{sen}$ is the estimated standard error of ${\hat{\theta }}_{sen}$. The estimate of ${\hat{\sigma }}_{sen}$ based on a restricted maximum likelihood estimation approach [3, 19, 20] is used, and the associated form is ${\hat{\sigma }}_{sen}=\sqrt{(2{\hat{p}}_{01}-\delta_{sen}(\delta_{sen}+1))/n}$, where

(6) p^01=B2−8A−B4,with  A=δsenδsen+1n01n,  B=−θ^sen1−δsen−2n01n+δsen.

There are two reasons for using this estimate instead of some other estimates [2]. First, it has been shown to perform well [8, 20]. Second, it is applicable to a 2 × 2 contingency table with off-diagonal zero cells. We are going to consider the exact approaches by enumerating all possible tables with some of them having zero cells in off-diagonals. The traditional estimate for *σ*_sen_ does not provide a reasonable estimate of variance for such tables.

The test statistic for sensitivity in (5) follows a normal distribution asymptotically. The null hypothesis *H*_0_:  *θ*_sen_ ≤ −*δ*_sen_ would be rejected if the test statistic *Z*_sen_ in (5) is greater than or equal to *z*_*α*_, where *z*_*α*_ is the upper *α* percentile of the standard normal distribution.

As mentioned by many researchers, the asymptotic approach has unsatisfactory type I error control especially in small or medium sample settings. An alternative is an exact approach by enumerating all possible tables for a given total of sample sizes. The first exact unconditional approach considered here is a method based on maximization (referred to as the *M* approach) [15]. The *p* value of this approach is calculated as the maximum of the tail probability. In this approach, the worst possible value for the nuisance parameter is found in order to calculate the *p* value, where **N**_obs_ is the observed data of **N**. The tail set based on the test statistic *Z*_sen_ for this approach is

(7) $$\begin{array}{c} R_{Z_{sen}}\left(\;N_{\text{obs}}\;\right)=\left\{\;N;Z_{sen}\left(\;N\;\right)\geq Z_{sen}\left(\;N_{\text{obs}}\;\right)\;\right\}. \end{array}$$

It is easy to show that (*n*_10_, *n*_01_∣*n*) follows a trinomial distribution with parameters (*n*; *p*_10_, *p*_01_). Then, the *M* *p* value is expressed as

(8) $$\begin{array}{c} P_{M}\left(\;N_{\text{obs}}\;\right)=\underset{p_{01}\in \Theta }{\max}\underset{N\in R_{Z_{sen}}\left(N_{\text{obs}}\right)}{\sum }\mathrm{Pr}\left(\;n_{10},n_{01};p_{01}\;\right), \end{array}$$

where Θ = (*δ*_sen_, min(1, (1 + *δ*_sen_)/2)) is the search range for the nuisance parameter *p*_01_ and Pr⁡(*n*_10_, *n*_01_; *p*_01_) = (*n*!/*n*_10_!*n*_01_!(*n* − *n*_10_ − *n*_01_)!)(*p*_01_ − *δ*_sen_)^*n*_10_^ *p*_01_^*n*_01_^(1 − 2*p*_01_ + *δ*_sen_)^*n*−*n*_10_−*n*_01_^ is the probability density function for a trinomial distribution.

The *M* approach could be conservative when the actual type I error is much less than the test size [5, 9]. To overcome this disadvantage of exact unconditional approaches, Lloyd [21] proposed a new exact unconditional approach based on estimation and maximization (referred to as the *E* + *M* approach). The first step in this approach is to compute the *p* value for each table based on the estimation approach [16], also known as parametric bootstrap. We refer to this approach as the *E* approach. The nuisance parameter in the null likelihood is replaced by the maximum likelihood estimate and the *E* *p* value is calculated as

(9) $$\begin{array}{c} P_{E}\left(\;N_{\text{obs}}\;\right)=\underset{N\in R_{Z_{sen}}\left(N_{\text{obs}}\right)}{\sum }\mathrm{Pr}\left(\;n_{10},n_{01};{\hat{p}}_{01}\;\right). \end{array}$$

It should be noted that the *E* approach does not guarantee type I error rate. Once the *E* *p* values are calculated for each table, they will be used as a test statistic in the next *M* step for the *p* value calculation. The *E* + *M* *p* value is then given by

(10) $$\begin{array}{c} P_{E+M}\left(\;N_{\text{obs}}\;\right)=\underset{p_{01}\in \Theta }{\max}\underset{N\in R_{E}\left(N_{\text{obs}}\right)}{\sum }\mathrm{Pr}\left(\;n_{10},n_{01};p_{01}\;\right), \end{array}$$

where *R*_*E*_(**N**_obs_) = {**N**; *P*_*E*_(**N**) ≤ *P*_*E*_(**N**_obs_)} is the tail set. The refinement from the *E* step in the *E* + *M* approach could possibly increase the actual type I error rate of the testing procedure which may lead to power increase for exact tests.

Monotonicity is an important property in exact testing procedures to reduce the computation time and guarantee that the maximum of the tail probability is attained at the boundary for noninferiority hypotheses. Berger and Sidik [22] showed that monotonicity is satisfied for paired data for testing one-sided hypothesis based on the NcNemar test. Most importantly, the dimension of nuisance parameters is reduced from two to one [17]. We provide the following theorem to show the monotonicity of the test statistic *Z*_sen_.

**Theorem 1 .**

Monotonicity property is satisfied for *Z*_*sen*_ under the null hypothesis: *Z*_*sen*_(*n*_10_, *n*_01_ + 1) ≤ *Z*_*sen*_(*n*_10_, *n*_01_) and *Z*_*sen*_(*n*_10_, *n*_01_) ≤ *Z*_*sen*_(*n*_10_ + 1, *n*_01_).

**Proof**

Let *x*_1_ = *n*_10_ and *x*_2_ = *n*_10_ + 1. For a given *n*_01_,

(11) Zsenx1−Zsenx2θ^senx1σ^senx2−θ^senx2σ^senx1+δsenσ^senx2−δsenσ^senx1σ^senx1σ^senx2=σ^senx2−σ^senx1θ^senx1+δsen+σ^senx1θ^senx1−θ^senx2σ^senx1σ^senx2=σ^senx2−σ^senx1θ^senx1+δsen−σ^senx1/nσ^senx1σ^senx2.

Under the null hypothesis, ${\hat{\theta }}_{sen}(x_{1})+\delta_{sen}\leq 0$. In order to show *Z*_sen_(*x*_2_) ≥ *Z*_sen_(*x*_1_), we only need to prove that ${\hat{\sigma }}_{sen}(x_{2})-{\hat{\sigma }}_{sen}(x_{1})\geq 0$. From (6), we know that

(12) $$\begin{array}{c} {\hat{p}}_{01}=\frac{\sqrt{B^{2}-8A}-B}{4}=\frac{-8A}{4\left(\sqrt{B^{2}-8A}+B\right)}, \end{array}$$

where *A* and *B* are given in (6). It is obvious that *B* is a decreasing function of *n*_10_ and *A* is a positive constant number when *n*_01_ is fixed and ${\hat{p}}_{01}$ is an increasing function of *n*_10_, which leads to ${\hat{\sigma }}_{sen}(x_{2})-{\hat{\sigma }}_{sen}(x_{1})\geq 0$. It follows that *Z*_sen_(*x*_2_) ≥ *Z*_sen_(*x*_1_).

For a given *n*_10_, similar proof will lead to a result of *Z*_sen_(*n*_10_, *n*_01_ + 1) ≤ *Z*_sen_(*n*_10_, *n*_01_).

The probability of the tail set for either the *M* approach or the *E* + *M* approach has two nuisance parameters, *p*_01_ and *p*_10_. Applying the theorem for the monotonicity property, type I error of the test occurs on the boundary of the two-dimensional nuisance parameter space, *p*_01_ = *p*_10_. Therefore, there is only one nuisance parameter, *p*_01_, in the definition of the two exact *p* values.

For testing the specificity, the asymptotic approach, the *M* approach, the *E* approach, and the *E* + *M* approach can be similarly applied to test the hypotheses *H*_0_:  *θ*_spe_ ≤ −*δ*_spe_ against *H*_*a*_:  *θ*_spe_ > −*δ*_spe_. The test statistic [3, 19, 20] would be

(13) $$\begin{array}{c} Z_{spe}=\frac{{\hat{\theta }}_{spe}+\delta_{spe}}{{\hat{\sigma }}_{spe}}, \end{array}$$

where ${\hat{\sigma }}_{\text{spe}}=(2{\hat{q}}_{10}-\delta_{\text{spe}}(\delta_{\text{spe}}+1))/n$ is the estimated standard error of ${\hat{\theta }}_{\text{spe}}$, ${\hat{q}}_{10}=(\sqrt{D^{2}-8C}-C)/4$, *C* = *δ*_spe_(*δ*_spe_ + 1)*m*_10_/*m*, and $D=-{\hat{\theta }}_{\text{spe}}(1-\delta_{\text{spe}})-2(m_{10}/m+\delta_{\text{spe}})$. Under the null hypothesis, one can show that the monotonicity of *Z*_spe_ is in a similar way to *Z*_sen_.

When there are two diagnostic tests available, we may want to simultaneously confirm the noninferiority of sensitivity and specificity for the two tests. The population from the diseased group and the nondiseased group can be reasonably assumed to be independent of each other. Then, the joint probability is a product of two probabilities:

(14) Pr⁡N,M ∣ N∈RNobs,M∈RMobs=Pr⁡N ∣ N∈RNobsPr⁡M ∣ M∈RMobs,

where *R* is the rejection region. Let *α*_sen_ and *α*_spe_ be the significance levels for testing sensitivity and specificity separately. We can reject the compound null hypothesis *H*_0_:  *θ*_sen_ ≤ −*δ*_sen_ or *θ*_spe_ ≤ −*δ*_spe_ at the significance level of *α* when the sensitivity null hypothesis is rejected at the level of *α*_sen_ and the specificity null is rejected at the level of *α*_spe_, where *α*_sen_ *α*_spe_ = *α*. For simplicity, we assume $\alpha_{sen}=\alpha_{\text{spe}}=\sqrt{\alpha }$.

## 3. Numerical Study

We already know that both the asymptotic approach and the *E* approach do not guarantee type I error rate; however, it is still interesting to compare type I error control for the following four approaches: (1) the asymptotic approach, (2) the *E* approach, (3) the *M* approach, and (4) the *E* + *M* approach. We select three commonly used values of *δ*_sen_ and *δ*_spe_, 0.05, 0.1, and 0.2. For each configuration of *δ*_sen_ and *δ*_spe_, actual type I error rates are presented in Table 2 for sample size *n* = *m* = 20 and in Table 3 for sample size *n* = *m* = 50 at the significance level of *α* = 0.05. It can be seen from both tables that the asymptotic approach generally has inflated type I error rates. Both the *M* approach and the *E* + *M* approach are exact tests and respect the test size as expected. Although the *E* approach does not guarantee type I error rate, the performance of the *E* approach is much better than the asymptotic approach regarding the type I error control. Even for large sample size, the *M* approach is still conservative. The *E* + *M* approach has an actual type I error rate which is very close to the nominal level when *n* = *m* = 50.

The asymptotic approach will not be included in the power comparison due to inflated type I error rates. We include the *E* approach in the power comparison with the *M* approach and the *E* + *M* approach due to the good performance of type I error control in the *E* approach. The power is a function of four parameters: *p*_01_, *θ*_sen_, *q*_10_, and *θ*_spe_

**Figure 1:**
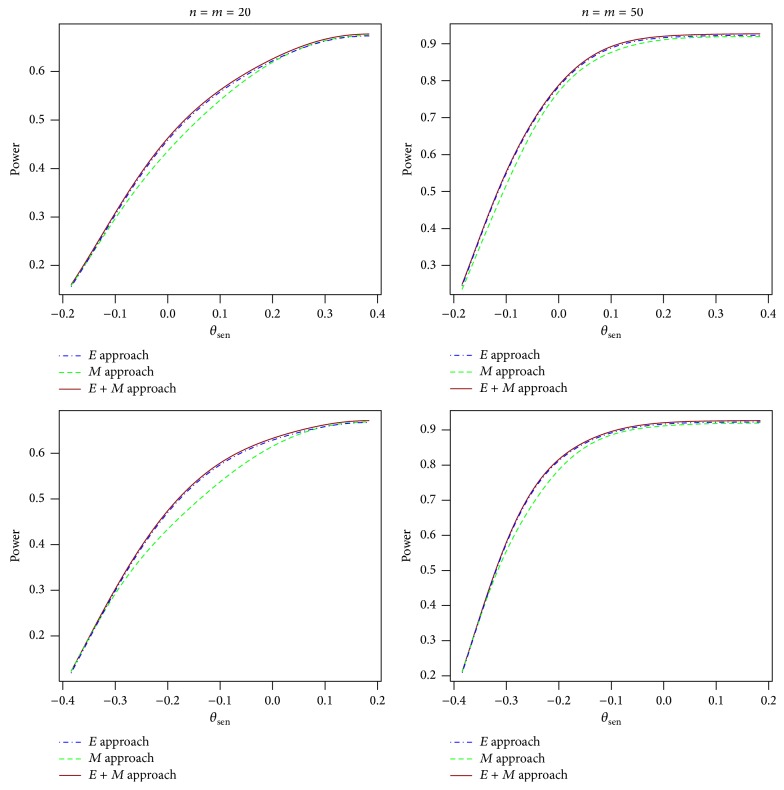
Power curves for the *E* approach, the *M* approach, and the *E* + *M* approach for balanced data with *θ*_spe_ = 0, *q*_10_ = 0.2, *p*_01_ = 0.3, *δ*_sen_ = 0.2, and *δ*_spe_ = 0.2 for the first row and *θ*_spe_ = 0, *q*_10_ = 0.2, *p*_01_ = 0.4, *δ*_sen_ = 0.4, and *δ*_spe_ = 0.2 for the second row.

**Figure 2:**
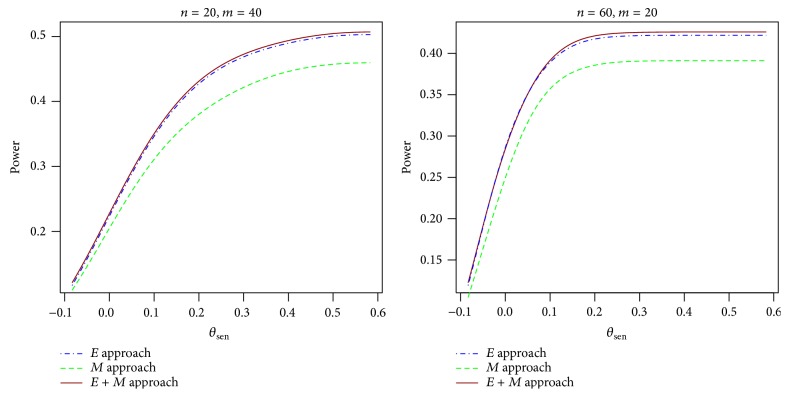
Power curves for the *E* approach, the *M* approach, and the *E* + *M* approach for unbalanced data with *θ*_spe_ = 0, *q*_10_ = 0.3, *p*_01_ = 0.2, *δ*_sen_ = 0.1, and *δ*_spe_ = 0.1.

(15) Powerϕ∑N∈RsenPr⁡n10,n01;p01,θsen·∑M∈RspePr⁡m10,m01;q10,θspe,

where *ϕ* = *E*, *M* and *E* + *M* approaches and *R*_sen_ and *R*_spe_ are the rejection region for the diseased group and the nondiseased group at a significance level of $\sqrt{\alpha }$ based on the *ϕ* approach. Given the two parameters *q*_10_ and *θ*_spe_ in the nondiseased group, the power is a function of *θ*_sen_ for a given *p*_01_. We compared multiple configurations of the parameters. Typical comparison results for balanced data are presented in Figure 1. The power difference between the *E* approach and the *E* + *M* approach is often negligible and both are generally more powerful than the *M* approach. We also compared the power for unbalanced data with the ratio of sample size 1/2, 1/3, 2, and 3. Similar results are observed as compared to the balanced data; see Figure 2. We also observe similar results in comparing the power as a function of *θ*_spe_ for the given *θ*_sen_, *p*_01_, and *q*_10_.

## 4. An Example

Kao et al. [1] compared diagnostic tests to detect recurrent or residual NPC in the presence of a gold standard test. Simultaneous comparison of sensitivity and specificity is conducted between the CT test (*T*_1_) and the Tc-MIBI SPECT test (*T*_2_), with *n* = 11 and *m* = 25. The diagnostic results using these two tests are presented in Table 4. The sensitivity and specificity are 73% and 88% for the CT test and 73% and 96% for the Tc-MIBI SPECT test. The clinical meaningful difference in sensitivity and specificity is assumed to be *δ*_sen_ = 0.01 and *δ*_spe_ = 0.01, respectively. Four testing procedures are used to calculate the *p* value: (1) the asymptotic approach; (2) the *E* approach; (3) the *M* approach; and (4) the *E* + *M* approach. The *p* values based on the asymptotic, *E*, *M*, and *E* + *M* approaches are 0.0677, 0.0317, 0.0764, and 0.0418, respectively. Both the *E* approach and the *E* + *M* approach reject the null hypothesis at a 5% significance level, while the asymptotic approach and the *M* approach do not. It should be noted that the two tests have the same sensitivities which may contribute to the significant result even with a small difference between the two tests.

## 5. Discussion

In this paper, the asymptotic approach, the *E* approach, the *M* approach, and the *E* + *M* approach are considered for testing sensitivity and specificity simultaneously in the presence of a gold standard. Although the *E* approach does not guarantee type I error rate, it has good performance regarding type I error rate control and the difference between the *E* approach and the *E* + *M* approach is negligible. Since the computational time is not an issue for this problem and the *E* + *M* approach is an exact method, the *E* + *M* approach is recommended for use in practice due to the power gain as compared to the *M* approach.

Tang [9] has studied the *E* approach and the *M* approach for comparing sensitivity and specificity when combining two diagnostic tests. The *E* approach has been shown to be a reliable testing procedure. We would consider comparing the *E* + *M* approach with the *E* approach in this context as a future work. The intersection-union method may be considered for testing sensitivity and specificity [8].

## Figures and Tables

**Table 1:** Test results from two diagnostic tests when a gold standard exists.

Diagnostic result	Diseased group		Nondiseased group	
	*T*_2_ = 1	*T*_2_ = 0	*T*_2_ = 1	*T*_2_ = 0
*T*_1_ = 1	*n*_11_(*p*_11_)	*n*_10_(*p*_10_)	*m*_11_(*q*_11_)	*m*_10_(*q*_10_)
*T*_1_ = 0	*n*_01_(*p*_01_)	*n*_00_(*p*_00_)	*m*_01_(*q*_01_)	*m*_00_(*q*_00_)

**Table 2:** Actual type I error rates *n* = *m* = 20.

*δ*_sen_	*δ*_spe_	*A* approach	*M* approach	*E* approach	*E* + *M* approach
0.05	0.05	0.1285	0.0343	0.0499	0.0489
	0.1	0.0894	0.0380	0.0489	0.0490
	0.2	0.0877	0.0401	0.0479	0.0480

0.1	0.05	0.0894	0.0380	0.0489	0.0490
	0.1	0.0621	0.0421	0.0481	0.0492
	0.2	0.0610	0.0444	0.0470	0.0481

0.2	0.05	0.0877	0.0401	0.0479	0.0480
	0.1	0.0610	0.0444	0.0470	0.0481
	0.2	0.0599	0.0468	0.0460	0.0471

**Table 3:** Actual type I error rates *n* = *m* = 50.

*δ*_sen_	*δ*_spe_	*A* approach	*M* approach	*E* approach	*E* + *M* approach
0.05	0.05	0.0821	0.0300	0.0492	0.0498
	0.1	0.0731	0.0341	0.0489	0.0493
	0.2	0.0677	0.0356	0.0486	0.0498

0.1	0.05	0.0731	0.0341	0.0489	0.0493
	0.1	0.0650	0.0387	0.0486	0.0489
	0.2	0.0603	0.0404	0.0482	0.0494

0.2	0.05	0.0677	0.0356	0.0486	0.0498
	0.1	0.0603	0.0404	0.0482	0.0494
	0.2	0.0559	0.0422	0.0479	0.0499

**Table 4:** Results of CT and Tc-MIBI SPECT diagnoses of NPC in the presence of a gold standard.

Diagnostic result	Diseased group (NPC: +)		Nondiseased group (NPC: −)	
	CT: +	CT: −	CT: +	CT: −
Tc-MIBI SPECT: +	5	3	1	0
Tc-MIBI SPECT: −	3	0	2	22
